# Evaluation and mechanism of ammonia nitrogen removal using sediments from a malodorous river

**DOI:** 10.1098/rsos.172257

**Published:** 2018-03-21

**Authors:** Xing Chen, Xia Jiang, Wei Huang

**Affiliations:** 1 National Engineering Laboratory for Lake Pollution Control and Ecological Restoration, Chinese Research Academy of Environmental Sciences, Beijing, People's Republic of China; 2 State Key Laboratory of Environmental Criteria and Risk Assessment, Chinese Research Academy of Environmental Sciences, Beijing, People's Republic of China

**Keywords:** ammonia nitrogen, removal, malodorous, sediment, sorption

## Abstract

Malodorous rivers are among the major environmental problems of cities in developing countries. In addition to the unpleasant smell, the sediments of such rivers can act as a sink for pollutants. The excessive amount of ammonia nitrogen (NH_3_−N) in rivers is the main factor that causes the malodour. Therefore, a suitable method is necessary for sediment disposition and NH_3_−N removal in malodorous rivers. The sediment in a malodorous river (PS) in Beijing, China was selected and modified via calcination (PS-D), Na^+^ doping (PS-Na) and calcination–Na^+^ doping (PS-DNa). The NH_3_−N removal efficiency using the four sediment materials was evaluated, and results indicated that the NH_3_−N removal efficiency using the modified sediment materials could reach over 60%. PS-DNa achieved the highest NH_3_−N removal efficiency (90.04%). The kinetics study showed that the pseudo-second-order model could effectively describe the sorption kinetics and that the exterior activated site had the main function of P sorption. The results of the sorption isotherms indicated that the maximum sorption capacities of PS-Na, PS-D and PS-DNa were 0.343, 0.831 and 1.113 mg g^−1^, respectively, and a high temperature was favourable to sorption. The calculated thermodynamic parameters suggested that sorption was a feasible or spontaneous (Δ*G* < 0), entropy-driven (Δ*S* > 0), and endothermic (Δ*H* > 0) reaction.

## 1. Introduction

The malodorous phenomenon in urban rivers is becoming increasingly serious in developing countries [1,2]. Water bodies are highly polluted by organic pollutants, nutrients (e.g. nitrogen and phosphorus) and heavy metals. As one of the main indexes of malodorous rivers, ammonia nitrogen (NH_3_–N) can lead to eutrophication, weakening of the self-depuration capability of water and threats to public health [3,4]. The sediments of malodorous rivers act as a sink for intense anthropogenic nitrogen inputs, and thus will become a major source of NH_3_–N after the effective interception of pollutants discharged into river systems. Therefore, economical and efficient methods for sediment disposal in malodorous rivers and NH_3_–N removal in water are essential.

The main technologies for NH_3_–N removal include physical, chemical and biological methods, such as air stripping [5], membrane separation [6], electro-oxidation [7,8], electrocoagulation [9], nitrification [10] and anammox [11]. Although most of these technologies are used in different on-site solutions, such as nitrification, electrocoagulation and several experimental projects, these methods are difficult to apply to large-scale engineering applications, particularly in malodorous rivers. In addition, the large amounts of sediments in such rivers require efficient disposition, which is realized through resourceful utilization. At present, the main technology for river sediment treatment includes on-site and off-site technologies, such as artificial aeration [12], coagulation [13], bioremediation [14] and dredging of sediment landfills [15], dehydration after dredging [16] and sediment pyrolysis after dehydration [17]. However, the on-site technologies are unsuitable for large-scale malodorous rivers. Moreover, sediment reutilization from malodorous rivers exhibits low economic benefits and inadequate protection. Although off-site technologies, such as dehydration or pyrolysis, are commonly used to dispose of malodorous river sediments, products from sediment reutilization exhibit no competitiveness compared with products from similar fields.

Sorption has been an effective method for NH_3_–N removal due to its simple process and high efficiency [18]. Thus, exploring new efficient materials for sorption has become a research focus. Natural absorbent materials always exhibit deficiencies in their structure and characteristics. To obtain more efficient effects, natural materials are typically modified via acid, alkali, salt, thermal, surfactant and microwave treatments [19]. The sediments in malodorous rivers are sources of pollutants and potential material resources of the pollutants. The Liangshui River (116°27′40.79^″^ N, 39°49′40^″^ E) is located in Beijing City, China and has a length of 64.8 km. With the development of the urban economy and the improvement of living standards, large amounts of contaminants and domestic garbage have been discharged into the river, which has resulted in serious malodour. The pollution from the sediment of Liangshui River is becoming increasingly serious.

This study aims to evaluate NH_3_−N removal efficiency using modified sediments from a malodorous river and to analyse the mechanism of NH_3_−N sorption. The modified sediment materials may be used to control eutrophication according to nitrogen removal. This study provides new insights into NH_3_−N removal and sediment reutilization from malodorous rivers.

## 2. Material and methods

### 2.1. Sediment sampling and sediment materials

Raw sediment samples were collected from the Liangshui River, a malodorous river located in Beijing, China, in February 2017. The samples were immediately brought to the laboratory, freeze-dried, ground, and sifted through a 100-mesh (0.15 mm) sieve to obtain a uniform size. The treated sediments were washed with HCl solution (pH = 1) to remove surface ash and organic matter. They were then filtered, dried and labelled as PS. The treated sediments were used to prepare the modified sediment materials. PS samples were placed in a muffle furnace for 2 h at 800°C for calcination in aerobic conditions, which were used to avoid the carbonization phenomenon, and cooled calcined PS samples (called PS-D) were obtained. Then, 50 g PS and 50 g PS-D were mixed with 1000 ml 0.20 mol l^−1^ NaCl solution for modification and shaken for 24 h at room temperature (25 ± 2°C). The modified sediment samples were obtained via filtration and drying and were named PS-Na and PS-DNa.

### 2.2. Analysis of physico-chemical parameters

Surface area and pore volume measurements were obtained using a Micromeritics Tristar 3000 Surface Analyzer. The oxide contents of the sediment materials were determined using an X-ray fluorescence analyzer (S4 Explorer, Germany). Organic matter (OM) content was determined by the loss on ignition to constant mass (4 h) at 550°C. The pH of the sediment was measured in a 1:2.5 (w/v) mixture of sediment with deionized water [20]. Table 1 shows the main properties of sediments and water in Liangshui River, and table 2 shows the characteristics of four sediment materials.

**Table 1.** Physico-chemical properties (mean value ± standard deviation) of sediments and water (*N *= 7) in Liangshui River. TP, total phosphorus; TN, total nitrogen; OM, organic matter; DO, dissolved oxygen.

	TP (mg kg^−1^)	TN (mg kg^−1^)	OM (%)	water content (%)	pH
raw sediment	1845.3 ± 167.5	3892.1 ± 273.2	10.67 ± 1.23	75.22 ± 12.32	8.37 ± 0.71

	TP (mg l^−1^)	TN (mg l^−1^)	NH_3_–N (mg l^−1^)	DO (mg l^−1^)	pH
water	0.89 ± 0.08	9.31 ± 1.05	6.48 ± 0.55	0.4 ± 0.12	8.18 ± 0.66

**Table 2.** Physico-chemical properties of the sediment materials.

	surface property analysis		oxide content (%)					physical property	
samples	surface area (m^2^ g^−1^)	pore volume (cm^3^ g^−1^)	SiO_2_	Na_2_O	Al_2_O_3_	MgO	CaO	OM (%)	pH
PS	2.83	8.89 × 10^−3^	62.5	0.43	17.9	1.56	0.87	7.84	7.35
PS-Na	7.55	1.29 × 10^−2^	58	2.32	17.1	1.69	0.39	7.84	7.35
PS-D	10.66	3.58 × 10^−2^	62.6	0.46	17.4	2.02	0.62	5.89	7.39
PS-DNa	14.97	6.89 × 10^−2^	59.8	2.31	17.5	1.74	0.17	5.89	7.39

### 2.3. Sorption kinetics experiments

NH_3_−N sorption kinetics was examined in a solution with initial NH_3_−N concentrations of 20 mg l^−1^ at 15°C, 25°C, and 35°C. Then, 1 g each of dried PS, PS-Na, PS-D and PS-DNa were mixed with 50 ml NH_4_Cl solution. The samples were covered and constantly agitated in a shaker (220 r.p.m) at constant temperatures of 15°C, 25°C and 35°C. Suspensions were obtained from each flask at 13 time intervals (0, 1, 2, 4, 8, 10, 20, 40, 60, 120, 240, 360 and 480 min). The suspensions were centrifuged, filtered (0.45 µm) and analysed to determine NH_3_−N concentration using the Nessler reagent spectrophotometric method [19]. Three groups of parallel experiments were set up.

### 2.4. Sorption isotherm tests

The sorption isotherm of NH_3_−N was obtained in batch experiments. Sediment materials (1 g in triplicate) of PS, PS-Na, PS-D and PS-DNa were added to 50 ml NH_4_Cl solution with different concentrations (0, 1, 2, 5, 10, 20 and 50 mg l^−1^). The samples were continuously agitated in a shaker at a speed of 220 rpm at constant temperatures of 15°C, 25°C and 35°C, for 480 min. The suspensions were centrifuged, filtered (0.45 µm), and analysed to determine NH_3_−N concentration.

### 2.5. Thermodynamic parameters

Sediment materials (1 g) of PS, PS-Na, PS-N and PS-DNa were mixed into 50 ml NH_3_−N solutions with different initial concentrations (0, 1, 2, 5, 10, 20 and 50 mg l^−1^) at 15°C, 25°C and 35°C. Batch samples were shaken in a temperature-controlled shaker for 480 min. The thermodynamic parameters of NH_3_−N sorption, such as enthalpy (Δ*H*), Gibbs energy (Δ*G*) and entropy (Δ*S*), were determined by fitting linear equations into the thermodynamic data obtained under different concentrations. Three groups of parallel experiments were set up.

### 2.6. Data analysis

The NH_3_−N uptake amount *Q_t_* (mg g^−1^) in different samples at equilibrium was calculated as follows:

2.1 $$Q_{t}=(C_{0}-C_{t})\frac{V}{W}.$$

The NH_3_−N removal efficiency *η* (%) was calculated as follows:

2.2 $$\eta \%=\frac{(C_{0}-C_{t})}{C_{0}}\times 100\%,$$

where *C*_0_ (mg l^−1^) is the initial liquid phase NH_3_−N concentration, *C_t_* (mg l^−1^) is the blank corrected concentration of NH_3_−N at time *t*, *W* (g) is the amount of dried sediment materials and *V* (l) is the volume of NH_3_−N solution.

The sorption kinetics was described by the pseudo-first-order, pseudo-second-order and power function models as follows [19,21,22]:

2.3 $$\begin{array}{r} Q_{t}=Q_{e}(1-{\text{e}}^{-K_{1}t}), \end{array}$$

2.4 $$\begin{array}{r} \frac{t}{Q_{t}}=\frac{1}{K_{2}Q_{e}^{2}}+\frac{t}{Q_{e}} \end{array}$$

2.5 $$\begin{array}{rl} \;\text{and}\;Q_{t} & ={\text{at}}^{b}, \end{array}$$

where *Q_t_* and *Q*_e_ (mg g^−1^) are the uptake amounts of NH_3_−N adsorbed at time point *t* and at equilibrium, respectively. *K*_1_ (h^−1^) is the first-order kinetic rate constant, *K*_2_ (g mg^−1^ h^−1^) is the sorption rate constant of the pseudo-second-order kinetic model, and *a* and *b* are the power function models of the kinetic rate constant.

The sorption isotherms were fitted by the Langmuir and Freundlich models, and the equations of the isotherm parameters are shown as follows [23,24]:

2.6 $$Q_{e}=\frac{Q_{m}KC_{e}}{1+KC_{e}}$$

and

2.7 $$Q_{e}=K_{f}C_{e}^{n},$$

where *Q*_e_ and *Q*_m_ (mg g^−1^) are the adsorbed amounts of NH_3_−N at equilibrium and the maximum NH_3_−N uptake amount, respectively; *C*_e_ (mg l^−1^) is the NH_3_−N concentration in aqueous phase at equilibrium; *K* (l mg^−1^) is the affinity parameter; *K*_f_ (l g^−1^) is the sorption coefficient; and *n* is a constant used to measure sorption intensity or surface heterogeneity.

The thermodynamic parameters were analysed using the following equations [25,26]:

2.8 $$\begin{array}{r} K_{D}=\frac{C_{0}-C_{e}}{C_{e}}\times \frac{V}{m}, \end{array}$$

2.9 $$\begin{array}{r} \Delta G^{\circ }=-\text{RT}\ln(K_{D}) \end{array}$$

2.10 $$\begin{array}{r} \;\text{and}\;\text{ln}(K_{D})=-\frac{\Delta H^{\circ }}{RT}+\frac{\Delta S^{\circ }}{R}, \end{array}$$

where Δ*G°* (kJ mol^−1^) is the change in Gibbs free energy, Δ*S°* (kJ mol^−1^ K^−1^) is the change in entropy, Δ*H°* (kJ mol^−1^ K^−1^) is the change in enthalpy, *K*_D_ is the equilibrium constant (dimensionless), *R* (8.314 J mol^−1^ K^−1^) is the gas constant, *T* (K) is the absolute temperature, *C*_0_ (mg l^−1^) is the initial solution concentration, *C*_e_ (mg l^−1^) is the solution equilibrium concentration, *V* (ml) is the solution volume and *m* (g) is the gravel sand mass.

## 3. Result and discussion

### 3.1. Effects of temperature on the removal of NH_3_−N using different modified sediment materials

**Figure 1.**
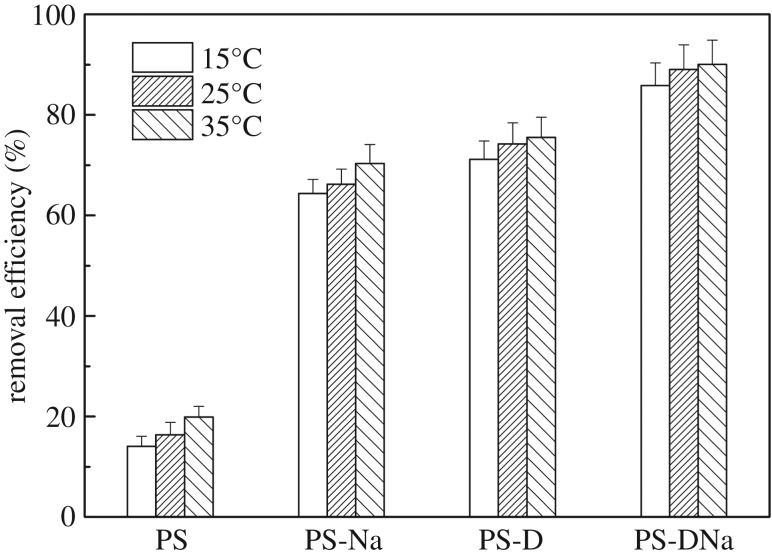
Effect of temperature on the removal of NH_3_−N using the four sediment materials.

Figure 1 shows the effect of temperature on the removal efficiency of NH_3_−N. The results illustrated that PS-DNa achieved the highest removal efficiency (average value: >90%), followed by PS-D (71%–76%) and PS-Na (64%–70%). The order of removal efficiency of NH_3_−N was PS-DNa > PS-D > PS-Na > PS. The NH_3_−N removal efficiency using the modified sediment materials increased rapidly when temperature increased from 15°C to 35°C. The modified materials demonstrated considerably higher NH_3_−N removal efficiency than the raw sediment samples. This observation could be explained by the fact that PS-DNa and PS-D had higher surface areas and pore volumes on the surface of the sediment owing to calcination. Table 2 shows that PS-DNa has the highest surface area of 14.97 m^2^ g^−1^. The amount of active sites on the surface of the sediment increased owing to sodium doping, and the sediment was loaded with a greater layer of active substances that could change its physico-chemical properties. Therefore, the NH_3_−N removal efficiency that used PS-Na and PS-DNa dramatically improved. The removal efficiency of the four sediment materials exhibited a rising trend when temperature increased from 15°C to 35°C, thereby indicating that increasing the temperature within limits could intensify molecular motion, accelerate diffusion rate and enhance the opportunity of $\text{N}{{\text{H}}_{4}}^{+}$ to impact on the sediment surface. In addition, $\text{N}{{\text{H}}_{4}}^{+}$ with high energy adsorbed on the surface of particles improved removal efficiency under high temperatures [27,28]. Previous studies indicated that natural zeolite had high NH_3_–N sorption capacity, and researchers focused on sorbents based on natural or modified zeolites [19,29]. The results indicated that PS-DNa had a similar removal efficiency of NH_3_–N (>90%) to the natural or modified zeolite (the highest value of 99.8%) [19,30]. In addition, PS-DNa had greater economic benefits than the natural or modified zeolite, and therefore PS-DNa might be a potential material for NH_3_–N removal.

### 3.2. Sorption kinetics of sediment materials

**Figure 2.**
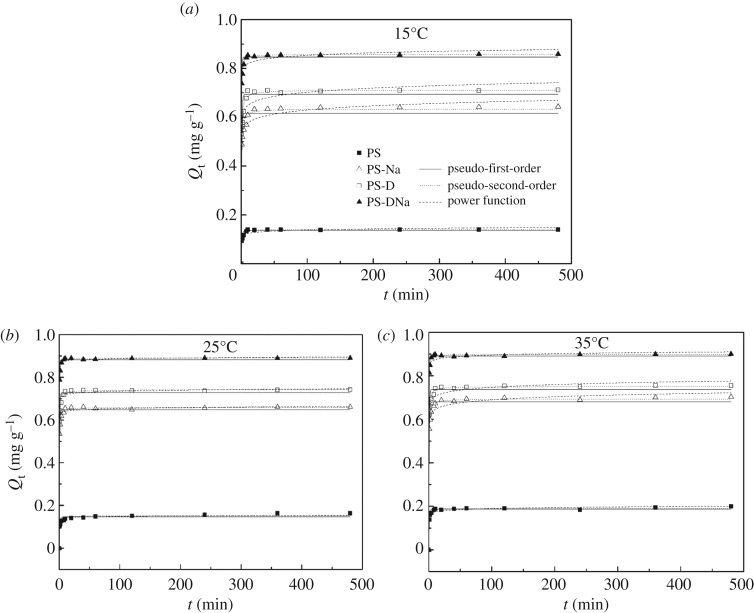
Sorption kinetics of NH_3_−N on the four sediment materials at various temperatures: (*a*) 15°C, (*b*) 25°C and (*c*) 35°C.

Figure 2 and table 3 show the kinetics of NH_3_−N sorption on the four sediment materials, along with the curves predicted from the pseudo-first-order, pseudo-second-order and power function kinetic models (equations (2.3), (2.4) and (2.5)). The results indicated that the order of NH_3_−N uptake capacity of the four modified sediment materials was PS-DNa > PS-D > PS-Na > PS, and the modified sediment samples had the highest values of *Q*_e_ at 35°C (0.891 mg g^−1^). NH_3_−N sorption on the four modified materials was initially fast and then rose rapidly during the first few minutes (0–10 min), but slowed down immediately after the initial uptake. The amount of NH_3_−N sorption tended toward equilibrium at approximately 40 min. The rapid original sorption could be attributed to physical sorption mechanisms, such as electrostatic interactions, which led to the adhesion of $\text{N}{{\text{H}}_{4}}^{+}$ on the material surface during the initial stage. Thereafter, the decreasing sorption rate could be interpreted as a ligand exchange [31].

**Table 3.** Fitting kinetics and mechanism parameters of NH_3_−N sorption on the four sediment materials according to the pseudo-first-order, pseudo-second-order and power function models at three different temperatures.

		pseudo-first-order model			pseudo-second-order model			power function model		
*T*	samples	*Q*_e_ (mg g^−1^)	*K*_1_ (min^−1^)	*R*^2^	*Q*_e_ (mg g^−1^)	*K*_2_ (g mg^−1^ min^−1^)	*R*^2^	*a*	*b*	*R*^2^
15°C	PS	0.140 ± 0.003	0.891 ± 0.110	0.9627	0.141 ± 0.001	12.044 ± 1.212	0.9911	0.111 ± 0.005	0.047 ± 0.011	0.9352
	PS-Na	0.617 ± 0.011	1.331 ± 0.207	0.9578	0.634 ± 0.007	4.170 ± 0.573	0.9888	0.526 ± 0.013	0.039 ± 0.006	0.9790
	PS-D	0.695 ± 0.010	1.231 ± 0.142	0.9742	0.711 ± 0.005	3.660 ± 0.320	0.9954	0.599 ± 0.020	0.035 ± 0.008	0.9646
	PS-DNa	0.846 ± 0.006	1.924 ± 0.164	0.9928	0.860 ± 0.002	6.722 ± 0.351	0.9994	0.787 ± 0.013	0.018 ± 0.004	0.9898
25°C	PS	0.147 ± 0.004	0.91 ± 0.169	0.9197	0.152 ± 0.003	9.925 ± 1.790	0.9696	0.136 ± 0.001	0.020 ± 0.008	0.7217
	PS-Na	0.649 ± 0.006	1.602 ± 0.143	0.9889	0.660 ± 0.002	6.231 ± 0.387	0.9986	0.628 ± 0.003	0.009 ± 0.001	0.8777
	PS-D	0.728 ± 0.007	1.53 ± 0.145	0.9866	0.742 ± 0.002	5.015 ± 0.276	0.9988	0.703 ± 0.002	0.010 ± 0.003	0.8631
	PS-DNa	0.883 ± 0.004	2.14 ± 0.138	0.9968	0.892 ± 0.002	8.545 ± 0.453	0.9996	0.867 ± 0.001	0.005 ± 0.001	0.9469
35°C	PS	0.187 ± 0.002	1.209 ± 0.128	0.9781	0.191 ± 0.001	13.338 ± 1.346	0.9938	0.160 ± 0.005	0.037 ± 0.008	0.9662
	PS-Na	0.680 ± 0.008	1.526 ± 0.167	0.9821	0.694 ± 0.003	5.143 ± 0.366	0.9979	0.603 ± 0.013	0.029 ± 0.005	0.9849
	PS-D	0.737 ± 0.008	1.689 ± 0.186	0.9848	0.751 ± 0.003	5.679 ± 0.470	0.9977	0.669 ± 0.013	0.025 ± 0.005	0.9867
	PS-DNa	0.891 ± 0.004	2.362 ± 0.149	0.9976	0.899 ± 0.002	10.751 ± 0.809	0.9995	0.854 ± 0.010	0.010 ± 0.003	0.9948

Table 3 shows that the kinetic parameters of NH_3_−N sorption derived from the four modified sediment materials are acquired through nonlinear fitting with the three models. The correlation coefficients (*R*^2^) demonstrated that the pseudo-second-order model achieved better fitting data (*R*^2^ > 0.96) than the pseudo-first-order and power function kinetic models. The fitting data showed that PS-DNa exhibited a relatively high equilibrium sorption capacity at different temperatures and reached its maximum value of 0.485 mg g^−1^ at 35°C. In addition, table 3 illustrates the change in the sorption rate constant *K*_2_. When ambient temperature is high, the *K*_2_ value is large. The result indicated that the crystal structure, surface area and pore volume of the sediment materials changed due to calcination or sodium doping, which improved NH_3_−N sorption on the surface of materials. Furthermore, sodium demonstrated greater capability to complex with the surface activity site of material particles and reacted more easily with the oxygen-containing functional groups when temperature was rising. Therefore, the modified sediment materials of PS-DNa have higher *K*_2_ values than those of PS-D and PS-Na.

### 3.3. Fitting of sorption isotherms

**Figure 3.**
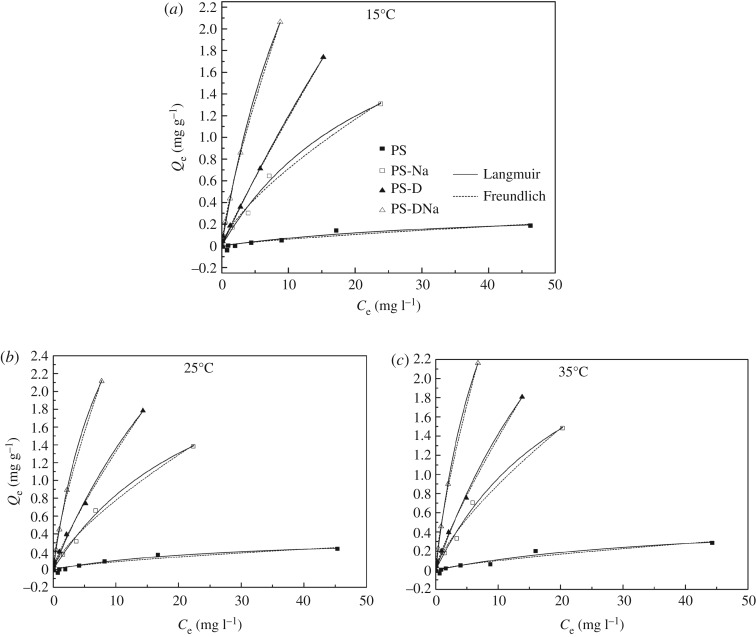
Sorption isotherms of NH_3_−N on the four sediment materials at different temperatures: (*a*) 15°C, (*b*) 25°C and (*c*) 35°C.

In this study, the isotherm data were fitted by the Langmuir and Freundlich models (equations (2.6) and (2.7)). Figure 3 and Table 4 show the results of the NH_3_−N sorption isotherm experiments. Both the Langmuir and Freundlich models could fit the data and estimate model parameters according to the correlation coefficients (*R*^2^). However, the Langmuir model can describe the NH_3_−N sorption isotherm better than the Freundlich model. The Langmuir sorption affinity parameter (*K*) and the maximum adsorption capacity (*Q*_m_) of the four materials appeared as continuous rising trends with increasing temperature, and the values for PS-DNa reached 4.986 l mg^−1^ and 1.113 mg g^−1^ at 35°C. At the same temperature, the *K* and *Q*_m_ of PS-DNa reached higher values than those of the other samples. The sorption capacity of the four materials increased with rising equilibrium concentration, which could be interpreted as the equilibrium concentration of the solution being closely related to the surface coverage density of the adsorbent. Under the condition of high equilibrium concentration, a large number of active sites on the surface of the material particles were occupied by the adsorbate, and the driving force of adsorption decreased and finally reached saturation state [32]. An increasing temperature could accelerate the diffusion rate, and a higher amount of high-energy $\text{N}{{\text{H}}_{4}}^{+}$ was adsorbed onto the surface of particles. In addition, the value of *n* obtained by the Freundlich model can reflect the strength of sorption capacity; when *n* is small, the adsorbate can be easily adsorbed by the materials [33]. PS-DNa achieved a higher *K*_f_ value than the other sediment materials, which indicated that PS-DNa had a higher sorption distribution coefficient and greater capacity to combine with NH_3_−N (table 4).

**Table 4:** Langmuir and Freundlich isotherm parameters of NH_3_−N sorption on the four sediment materials.

		Langmuir			Freundlich		
temperature	samples	*Q*_m_ (mg g^−1^)	*K* (l mg^−1^)	*R*^2^	*N*	*K*_f_ (l g^−1^)	*R*^2^
15°C	PS	0.024 ± 0.022	0.366 ± 0.188	0.8764	0.011 ± 0.008	0.768 ± 0.215	0.8375
	PS-Na	0.140 ± 0.010	2.683 ± 0.385	0.9903	0.138 ± 0.022	0.712 ± 0.053	0.9865
	PS-D	0.411 ± 0.005	12.416 ± 5.414	0.9970	0.146 ± 0.012	0.915 ± 0.032	0.9975
	PS-DNa	0.695 ± 0.012	5.651 ± 0.698	0.9974	0.379 ± 0.013	0.781 ± 0.017	0.9990
25°C	PS	0.035 ± 0.019	0.389 ± 0.109	0.9314	0.018 ± 0.009	0.692 ± 0.152	0.8838
	PS-Na	0.289 ± 0.009	0.009 ± 0.424	0.9920	0.145 ± 0.021	0.729 ± 0.050	0.9887
	PS-D	0.628 ± 0.008	6.190 ± 1.365	0.9957	0.197 ± 0.014	0.827 ± 0.028	0.9975
	PS-DNa	0.923 ± 0.012	4.603 ± 0.308	0.9984	0.473 ± 0.021	0.734 ± 0.024	0.9976
35°C	PS	0.025 ± 0.016	0.561 ± 0.210	0.9272	0.018 ± 0.01	0.736 ± 0.151	0.9005
	PS-Na	0.343 ± 0.011	3.197 ± 0.487	0.9907	0.166 ± 0.025	0.731 ± 0.055	0.9867
	PS-D	0.831 ± 0.007	6.054 ± 1.094	0.9968	0.208 ± 0.011	0.822 ± 0.022	0.9984
	PS-DNa	1.113 ± 0.013	4.986 ± 0.368	0.9984	0.517 ± 0.018	0.748 ± 0.020	0.9985

### 3.4 Thermodynamic parameters

**Figure 4.**
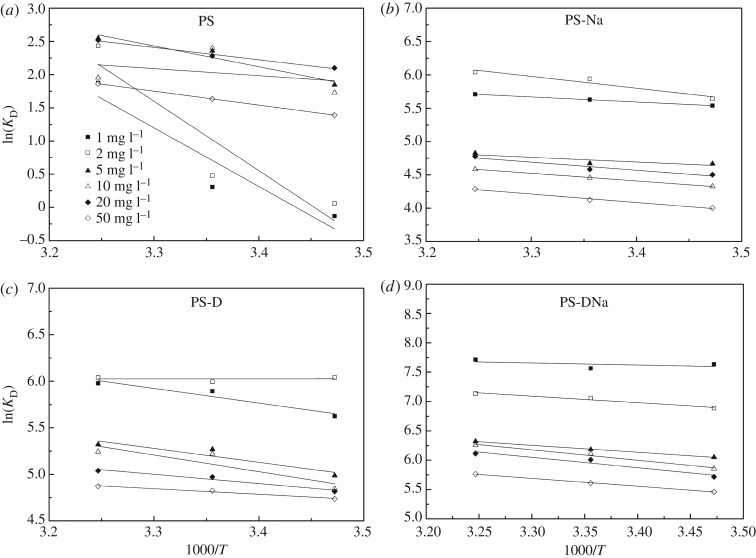
Thermodynamic analysis of NH_3_−N sorption onto (*a*) PS, (*b*) PS-Na, (*c*) PS-D and (*d*) PS-DNa.

We acquired the values of Δ*H*^0^ and Δ*S*^0^/*R* (figure 4) after fitting ln*K*_D_ and 1000/*T* according to equation (2.10). The thermodynamic parameters were calculated, and the results are presented in table 5. Δ*G*^0^ can be calculated using equation (2.9). At the same temperature, the Δ*G*^0^ values of the modified sediment materials are lower than that of the raw sediment. This condition indicates that NH_3_−N is adsorbed first using the modified sediment materials compared with using raw sediment in the process of NH_3_−N removal using sediment materials. However, Δ*G*^0^ decreased as temperature increased, which indicated that the NH_3_−N sorption of the modified sediment materials was better at high temperatures [34]. The positive values of Δ*H*^0^ indicated that NH_3_−N sorption was an endothermic process. In addition, Δ*S*^0^ values were higher than 0, which indicated that NH_3_−N tended to be adsorbed onto the surface of sediment materials [35]. In general, entropy decreases when molecules are adsorbed onto the surface of solid materials. In the solute sorption process, the solute molecular degree of freedom dropped and the entropy of the modified sediment materials was lower than that of the raw sediment. Consequently, the sorption of NH_3_−N is a complex process, and the entropy effect is the driving force of the sorption process [19].

**Table 5:** Thermodynamic parameters of NH_3_−N sorption on the four sediment materials.

	PS					PS-Na				
			Δ*G*^0^ (kJ mol^−1^)					Δ*G*^0^ (kJ mol^−1^)		
*C*_0_ (mg l^−1^)	Δ*H*^0^ (kJ mol^−1^)	Δ*S*^0^ (kJ mol^−1^ K^−1^)	15°C	25°C	35°C	Δ*H*^0^ (kJ mol^−1^)	Δ*S*^0^ (kJ mol^−1^ K^−1^)	15°C	25°C	35°C
1	73.44	0.25	0.31	−0.76	−4.80	6.26	0.07	−13.26	−13.95	−14.62
2	86.88	0.30	−0.14	−1.18	−6.23	14.76	0.10	−13.51	−14.72	−15.47
5	26.01	0.11	−4.42	−5.84	−6.52	5.84	0.06	−11.18	−11.58	−12.36
10	8.80	0.05	−4.14	−5.95	−5.01	9.53	0.07	−10.37	−11.04	−11.75
20	15.47	0.07	−5.03	−5.65	−6.45	10.04	0.07	−10.78	−11.36	−12.23
50	17.43	0.07	−3.33	−4.05	−4.77	10.49	0.07	−9.59	−10.22	−10.98

	PS-D					PS-DNa				
			Δ*G*^0^ (kJ mol^−1^)					Δ*G*^0^ (kJ mol^−1^)		
*C*_0_ (mg l^−1^)	Δ*H*^0^ (kJ mol^−1^)	Δ*S*^0^ (kJ mol^−1^ K^−1^)	15°C	25°C	35°C	Δ*H*^0^ (kJ mol^−1^)	Δ*S*^0^ (kJ mol^−1^ K^−1^)	15°C	25°C	35°C
1	13.09	0.09	−13.47	−14.60	−15.31	2.86	0.07	−18.28	−18.74	−19.75
2	0.04	0.05	−14.46	−14.85	−15.47	9.27	0.09	−16.49	−17.49	−18.27
5	12.43	0.08	−11.94	−13.05	−13.62	9.96	0.08	−14.49	−15.32	−16.18
10	14.87	0.09	−11.60	−12.93	−13.43	14.81	0.10	−14.02	−15.14	−16.02
20	8.25	0.07	−11.53	−12.32	−12.90	14.71	0.10	−13.69	−14.88	−15.65
50	4.99	0.06	−11.34	−11.95	−12.48	11.17	0.08	−13.08	−13.90	−14.76

## 4. Conclusion

Solutions must be obtained to address problems of excessive NH_3_−N and the utilization of sediment resources in malodorous rivers. The sediment from a malodorous river was modified and used to remove NH_3_−N. The NH_3_−N removal efficiency was evaluated, and the NH_3_−N sorption mechanism was studied through kinetics, equilibrium and thermodynamic experiments. The results indicated that the calcination–sodium-doped materials achieved the highest NH_3_−N removal efficiency. The sediment sorption rate followed the pseudo-second-order model, and high temperatures favoured NH_3_−N uptake. Overall, the data that described the sorption isotherms were better fitted by the Langmuir model. The maximum P sorption capacities exhibited the following order: PS-DNa > PS-D > PS-Na > PS. PS-DNa yielded the highest value of 1.113 mg g^−1^. Sorption was feasible or spontaneous (Δ*G* < 0), randomly entropy-driven (Δ*S* > 0), and endothermic (Δ*H* > 0) according to the calculation of the thermodynamic parameters of the sediment materials. This study provides a new method for the utilization of sediment resources and a theoretical foundation for NH_3_−N removal.

## Data accessibility

Data are available on Dryad: http://dx.doi.org/10.5061/dryad.t85f7 [36].
